# Robotic Mitral Valve Repair: Impact of Experience on Results and Complex Mitral Disease Treatment

**DOI:** 10.3390/jcm13133744

**Published:** 2024-06-26

**Authors:** Antonio Lio, Marco Russo, Beatrice Sangiorgi, Francesca Nicolò, Ilaria Chirichilli, Francesco Irace, Federico Ranocchi, Francesco Musumeci

**Affiliations:** Department of Cardiac Surgery and Transplantation, S. Camillo Hospital, Circonvallazione Gianicolense 87, 00152 Rome, Italy; mar.russo1987@gmail.com (M.R.); beatrice.sangiorgi@unicampus.it (B.S.); nicolo_francy84@hotmail.it (F.N.); chirichilli@inwind.it (I.C.); francesco_irace@hotmail.it (F.I.); franocchi@scamilloforlanini.rm.it (F.R.); fr.musumeci@gmail.com (F.M.)

**Keywords:** mitral valve, mitral valve repair, robotic surgery, minimally invasive

## Abstract

**Background/Objectives**: Robotically assisted mitral valve (MV) surgery is the least invasive surgical approach to the MV. The aim of the present study is to report our experience with robotically assisted MV repair, trying to define how experience could impact on postoperative results. **Methods**: This is a retrospective study on 144 patients who underwent robotic MV repair from November 2011 to March 2023. Patients were divided in two groups: Group 1, including 39 patients (November 2011–January 2013) operated using the Da Vinci Si system, and Group 2, including 105 patients operated (February 2020–March 2023) using the new Da Vinci Xi system. **Results**: Mean age was 58 ± 10 years. Increased use of external aortic clamp was observed in Group 2. A significant reduction of surgical times was observed: cardiopulmonary bypass time was 155 ± 44 min in Group 1 and 121 ± 36 min in Group 2 (*p* = 0.002), whereas cross-clamp time was 112 ± 25 min in Group 1 and 68 ± 39 min in Group 2 (*p* < 0.001). In-hospital mortality was 0.7%, and 10-year survival was 96 ± 2%. Freedom from reoperation was 100%. A higher percentage of complex and most complex MV repairs were performed in Group 2 (36% in Group 1 vs. 52% in Group 2, *p* = 0.001). **Conclusions**: Robotic-assisted MV repair is associated with excellent results. Experience is a key element to overcome the limitations of this technology. Finally, the robotic platform could improve results in difficult MV repair.

## 1. Introduction

Robotically assisted mitral valve (MV) surgery is the least invasive surgical approach to the MV; it was introduced in the late 1990s. The primary goal was to improve the technical precision of less-invasive surgical MV repair [1]. Literature has shown excellent outcomes of robotic MV repair with high survival, excellent durability, and infrequent complications; benefits related with this kind of approach include a shorter in-hospital length of stay (LOS), reduced pain, improved cosmesis, reduced need for blood transfusions, and, consequently, a faster return to ordinary daily activities [2,3,4,5,6,7,8,9]. After initial enthusiasm in the early 2000s, robotic cardiac surgery did not grow as fast as expected; its main concerns regarded prolonged operative time, the quality of valve repair, and significant costs. The long learning curve is an important issue of robotic MV surgery. Literature suggests that in the beginning of the learning curve period only simple MV disease should be addressed [10,11,12]. However, with experience, moving to complex MV repairs, robotic surgery has shown results comparable with more simple operations with no increase in the need of reoperations [8]. In this setting, some authors have advocated the use of robotic surgery in all complex MV repair cases [8,13,14]. Recent literature has also shown that robotic surgery is related to potential benefits compared to other minimally invasive approaches used for MV disease treatment, such as minithoracotomy or ministernotomy. These benefits include a shorter in-hospital length-of-stay and faster return to daily life, with consequent benefits from a social point of view [14,15,16]. However, the value of this technology and its clinical benefits are questioned because it is associated with high costs. Although costs remain an important limitation, some studies have shown that they could be also reduced with experience [15,16,17]. The aim of the present study was to report our single-center experience with robotically assisted MV repair, describing differences observed between two study periods, trying to define how experience could impact on postoperative results.

## 2. Materials and Methods

A retrospective, observational, cohort study was undertaken of prospectively collected data on consecutive patients undergoing robotic MV repair at our institution between November 2011 and March 2023. Every patient signed an informed consent for anonymous data collection. All preoperative, operative, and postoperative data were entered in a local database, including several sections that were filled in by the anesthetists, cardiac surgeons, and perfusionists involved in the care of the patients. The sample comprised 144 patients. Strong contraindications for a robotic surgery were previous right thoracotomy, fixed pulmonary hypertension (>60 mmHg), right ventricular dysfunction, severe generalized peripheral artery disease, severe pulmonary dysfunction, severe liver dysfunction, significant bleeding disorder. Relative contraindications were previous sternotomy, reduced left ventricular function (<50%), chest deformity. All operations were performed with the da Vinci Surgical System (Intuitive Surgical, Inc., Sunnyvale, CA, USA); particularly, patients were divided in two groups: Group 1, including 39 patients operated between November 2011 and January 2013 using the Da Vinci Si system, and Group 2, including 105 patients operated between February 2020 and March 2023 using the new Da Vinci Xi system. From February 2013 to January 2020, the robotic program was suspended due to hospital administration reasons. All procedures were performed by the same surgeon. MV repair complexity was scored using Loulmet classification [13]: category I (simple) included cases of annuloplasty alone or repair of 1 leaflet segment (e.g., triangular excision-suture, placement of artificial chordae); category II (complex) included cases involving the repair of more than 1 segment on the same leaflet (e.g., sliding plasty, chordal transfer, papillary muscle repositioning, leaflet patch augmentation); category III (most complex) included cases of bileaflet repair or MAC excision and AV groove repair. The primary endpoints were operative mortality, defined as any death occurring within 30 days of operation or before discharge from the hospital, long-term survival, and freedom from reoperation. Secondary endpoints were postoperative outcomes and complexity of MV repair. Follow-up was complete at 100%.

### 2.1. Surgical Technique

The patient was placed in a supine position with an air sac under the right scapula, thereby elevating the right chest to achieve optimal exposure of the working field. Cardiopulmonary bypass (CPB) is generally established through a femoro-femoral platform; femoral vessels are exposed through a 2 cm transverse incision in the groin, and 2 single 5.0 polypropilene purse-string sutures are inserted on the artery and vein, respectively. Vessel cannulation was performed with the Seldinger technique; particularly, the vein cannulation was performed under transesophageal echocardiographic guidance using a bicaval view to ensure that the cannula was properly advanced into the superior vena cava. In case of bicaval cannulation, the right jugular vein was cannulated by anesthesiologist before starting the operation. Then, the working access was created with a 2 cm skin incision performed into the right chest at the level of the middle-axillary line; the 4th intercostal space (ICS) was entered, and a soft-tissue retractor was inserted into the minithoracotomy. Four dedicated robotic ports were inserted: the port for the video assistance was positioned into the 4th ICS, medially to the working access point. The second and the third ports, for the robotic arms, were positioned into the 3rd and 6th ICS, respectively. The last port access was positioned in the 4th ICS medially to the first, just next to the sternum, for the atrial retractor. After CPB establishment and pericardium opening, a 3.0 purse-string suture reinforced with a Teflon pledget was made on the ascending aorta for insertion of a combined Y-shaped vent/cardioplegia catheter. The aorta was clamped using either the EndoClamp (EndoClamp^®^ Intra-aortic Occlusion Device, Edwards Lifesciences Corp. Irvine, CA, USA) or the external Chitwood aortic clamp, inserted through the 2nd ICS, and cardioplegia was delivered as a single dose/shot (20 mL/kg) of cold crystalloid cardioplegia solution (Custodiol) into the aortic root. The MV was approached through the Sondergaard’s groove and exposed using the dedicated robotic left atrial retractor; after MV analysis, the repair was performed. Annuloplasty sutures were always tied using the Cor-Knot system (LSI Solutions, Victor, NY, USA). After left atrial closure and deairing, the aortic vent purse-string suture was tied with the Cor-Knot system. The patient was weaned from CPB, and the operation was ended in a standard fashion.

### 2.2. Statistical Analysis

Continuous data are presented as mean ± SD or as median with the interquartile range, and categorical data are expressed as percentages. Student’s *t*-test was used to compare continuous variables when normal distribution was present, as confirmed by the Kolmogorov–Smirnov test. The Mann–Whitney test was used for nonnormally distributed variables. For the Student’s unpaired *t*-test, the Levene test for variance equality was used, which was not significant at >0.05, and thus variance equality was assumed. Categorical variables were compared with the χ^2^ test. The Fisher exact test was used for small group sizes (*n* < 5). A Kaplan–Meier curve was generated to provide survival estimates at postoperative points in time. *p*-value of <0.05 was considered statistically significant. All statistical analyses were performed with SPSS 15.0 (SPSS, Inc., Chicago, IL, USA).

## 3. Results

### 3.1. Preoperative and Operative Data

Preoperative data are shown in Table 1. Mean age was 58 ± 10 years, with a higher percentage of male patients (91%). Mean EuroSCORE II and STS scores were 0.8 ± 0.5 and 0.6 ± 0.3, respectively. No significant difference existed between the two study groups in terms of comorbidities. Echocardiography data showed a significant higher rate of patients with anterior leaflet pathology in Group 2. Table 2 summarizes operative data. A transareolar approach was used in 14 patients (10%), with no difference between the two study groups. Additional jugular vein cannulation was performed in 50% of patients, with a significant higher percentage in the earlier experience (100% in Group 1 vs. 31% in Group 2, *p* < 0.001). The EndoClamp was used only in patients of Group 1 (72% of patients), whereas all cases of Group 2 were performed using an external Chitwood clamp. In Group 2, there was a significant reduction of surgical times: the CPB time was 155 ± 44 min in Group 1 and 121 ± 36 min in Group 2 (*p* = 0.002), whereas the cross-clamp time was 112 ± 25 min in Group 1 and 68 ± 39 min in Group 2 (*p* < 0.001). Only one patient required conversion to sternotomy due to bleeding from the ascending aorta.

### 3.2. Postoperative and Follow-Up Data

**Figure 1:**
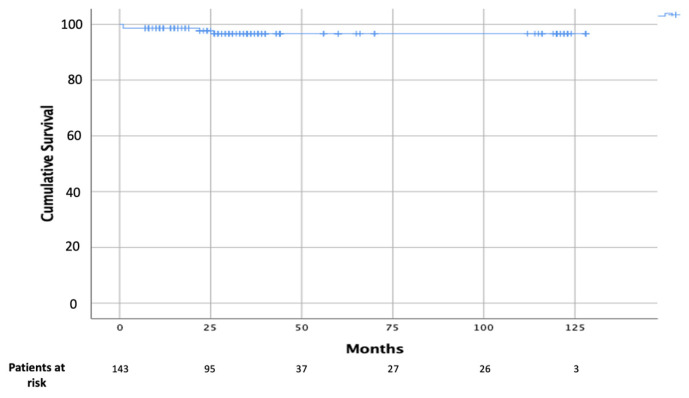
Kaplan–Meier curve of overall survival.

Postoperative data are shown in Table 3. Only one patient died (0.7%), due to sepsis and consequent multiorgan failure. Rate of postoperative complications was very low. Overall, 24 patients (16%) required blood transfusions, with a median blood unit value of one unit; in particular, the rate of transfusions was significant lower in Group 2 (33% in Group 1 vs. 10% in Group 2, *p* < 0.001). Median Intensive Care Unit (ICU) LOS was 24 h, and median in-hospital LOS was 8 days. Echocardiography performed at discharge showed no patient with more than mild MV regurgitation. Median and mean follow-up were 45 ± 38 and 32 (interquartile range: 19–56) months, respectively. In addition, 10-year survival was 96 ± 2% (Figure 1). Freedom from reoperation was 100%.

### 3.3. Mitral Valve Repair Complexity

**Figure 2:**
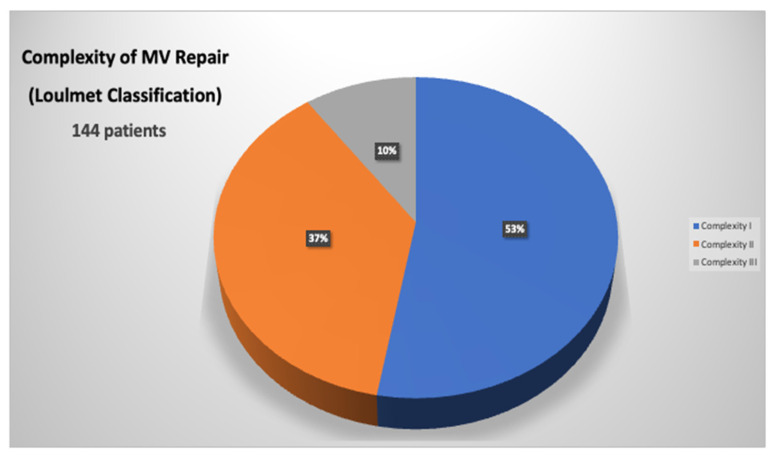
Distribution of MV complexity in all patients using Loulmet classification.

**Figure 3:**
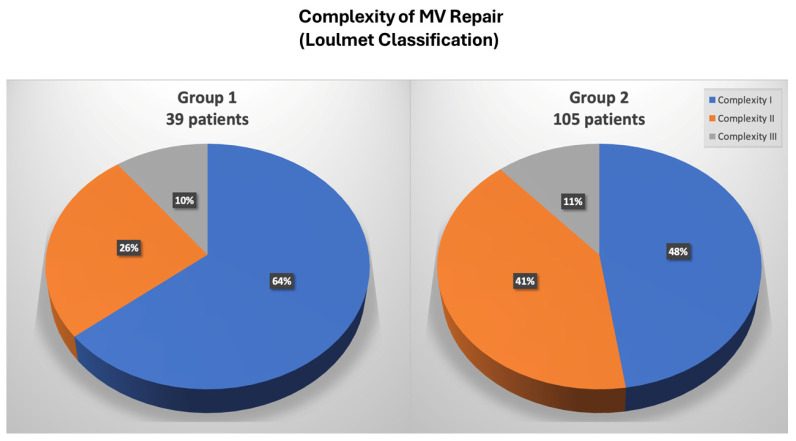
Distribution of MV complexity in Group 1 and Group 2 patients using Loulmet classification.

In Group 1 a significant higher use of neochordae was observed (65% in Group 1 vs. 14% in Group 2, *p* < 0.001), whereas in Group 2, there was a significant increase of complex resectional techniques, such as quadrangular resection and sliding plasty (Table 2). Simple (Category I) MV repairs were performed in 53% of patients, whereas complex (Category II) and most complex (Category III) were performed in 37% and 10% of patients, respectively (Figure 2). With experience, a significant increase in MV repair complexity was observed, with a higher percentage of Category II and III MV repairs in Group 2 (36% in Group 1 vs. 52% in Group 2, *p* = 0.001) (Figure 3).

## 4. Discussion

Minimally invasive cardiac surgery has dramatically evolved over the past two decades with the final goal of reducing invasiveness, post-operative complications, and hospital stays [18,19]. In this context, robotic surgery emerged as the most advanced technological method of minimally invasive MV repair; introduced in the late 1990s, it had the primary goal of improving the technical precision of MV reconstruction in a minimally invasive setting; however, many have challenged the use of robotic valve repairs, basing their concerns on inferior safety and outcomes, especially when dealing with a largely elective, low-risk population. The goal of robotic MV surgery from the inception was the exact replication of conventional MV repair to attain the known efficacy and durability outcomes that have been proven over the preceding decades. In this setting, data are present in literature regarding the excellent outcomes of this technology [2,3,4,5,6,7,8,9]. In 2015, Murphy et al. reported outcomes in 1257 consecutive cases of robotic MV surgery, of which 93% were MV repair, with a very low rate of mortality of 0.9%; trace or mild mitral regurgitation was found in over 98% of repaired valves and at a mean follow-up of 50 +/− 26 months, 3.8% patients required re-operation [4]. Gillinov et al. have reported their own experience on 1000 robotic mitral procedures with similar outcomes: in-hospital mortality was 0.1%, and stroke rate was 1.4%, which declined from 2% in the first 500 patients in the series to 0.8% in the second 500 patients. Successful MV repair rate was 99%, and 97.8% of these patients had mild or less mitral regurgitation at discharge [20]. Also, long-term results are encouraging. Roach et al. reported a freedom from all-cause mortality at 10-year follow-up of 91% in the isolated posterior leaflet group versus 87% in the anterior or bileaflet group; moreover, 10-year freedom from >2+ moderate regurgitation or reintervention was 91% for patients with isolated posterior leaflet prolapse versus 83% for anterior or bileaflet prolapse [21]. We have found similar results in our series, with a low rate of postoperative mortality (0.7%) and morbidity; long-term results were also excellent: 10-year survival of 96 ± 2% and 100% freedom from reoperation. However, the worldwide spread of this approach has been held back for several reasons, particularly the high costs and an apparently long learning curve [22,23,24]. Gillinov et al. have reported a reduction of CPB and cross-clamp times after approximately 200 cases [20]. However, more recent series have been able to demonstrate that the maturation of the learning curve could be mitigated, with a target of around 30 cases [24,25]. Another important result of our study is the progressive reduction of CPB and cross-clamp times after a relatively small sample of operated patients. Comparing two different study periods, we highlighted a significant reduction of surgical times: the CPB time was 155 ± 44 min in Group 1 and 121 ± 36 min in Group 2, whereas the cross-clamp time was 112 ± 25 min in Group 1 and 68 ± 39 min in Group 2. This result is obviously related to the learning curve effect, but considering the 7-year suspension of the robotic program, it could be related to the evolution of the robotic system itself. As Trento’s group has emphasized, the robotic device has enormously through three generations available of the da Vinci surgical system [26,27]. The most important improvements of the latest robotic platform included: 3D vision, depth perception (that allow a better intraoperative vision), and wristed instrumentation with enhanced ergonomics; all these changes have led to an improving of operative results. A significant issue of robotic surgery is represented by the increased costs. Although robotic MV repair was initially associated with higher total hospital resource use, costs have decreased significantly with experience and with the evolution of programmatic efficiencies. The total hospital cost associated with the use of robotic MV repair has now become like conventional open operation, particularly in high-volume centers [15,16,17]. Paul and colleagues reviewed data from 50,408 patients having had any mitral repair in the United States between 2008 and 2012 [28]. A comparison between 3145 robotic patients and all other repair operations was performed, using a propensity-matched analysis to reduce selection bias. The most important finding of the analysis was the absence of any difference in overall cost or complications between the two study groups. Moreover, the robotic group showed a slightly (*p* = 0.048) reduced in-hospital mortality. Particularly, cost reduction is mainly related to postoperative cost saving [15,16,17]. Mihaljevic et al. retrospectively compared MV repair performed by robotic approach with three other options (ministernotomy, right minithoracotomy, conventional sternotomy) by creating propensity-matched groups [16]. Authors have included in the analysis of operative costs all the robotic-specific instruments and procedure-specific disposables, such as double-lumen endotracheal tubes, external defibrillator patches, and special cannula for CPB. Direct technical operative costs were 37%, 37%, and 20% higher for robotic MV surgery than for operations performed through a complete sternotomy, partial sternotomy, or minithoracotomy, respectively; however, direct technical postoperative costs were 16%, 17%, and 10% lower for robotic surgery than for these three alternatives, resulting in total direct hospital technical costs that were 18%, 24%, and 12% higher, respectively. Moreover, another important finding was that return to work was earlier after robotic MV repair than after the alternative approaches (29%, 38%, and 17%). Suri et al. have conducted a study on 185 propensity-matched pairs of conventional MV repair surgery and robotic MV repair surgery; they found that robot surgery was associated with shorter ICU LOS and hospital LOS [15]. Moreover, with implementation of systems innovations (after July 2009) the cost of robotic surgery has significantly reduced, reaching a level comparable to that of open surgery (median: USD 30,606 vs. USD 31,310). Intraoperative implementations that have guaranteed a significant cost reduction were external aortic cross-clamping and the antegrade delivery of cardioplegia. In our study, although costs were not evaluated in the analysis, we have demonstrated that, with experience, it was possible to shift from an endo-aortic cross-clamping system to an external cross-clamp, leading to cost saving. In this setting, routinely use of a single venous cannulation (instead of a double jugular-femoral cannulation), that has been achieved in the late experience, is another important goal to reduce costs. Another important field of interest of robotic surgery is complex MV disease treatment: literature data have shown that robotic technology makes it possible to address the entire spectrum of MV pathology including mitral annular calcification (MAC), a common contraindication to repair. Loulmet et al. have published a series on 64 patients with mitral annular calcification undergoing robotic mitral surgery, which accounted for just under 13% of their robotic case volume over a 6-year period [29]. Operative outcomes were excellent, with a reported rate of MV repair of 97%; no patient showed at discharge residual mitral regurgitation more than trace or mild. Authors have also reported encouraging postoperative outcomes, with a 30-day mortality rate of 3.1% and no stroke. The same authors have also analyzed their whole robotic mitral experience over the same period on nearly 500 patients: 24% of cases were considered most complex MV pathologies (bileaflet repair or atrioventricular groove reconstruction for MAC), finding longer cross clamp time and CPB times, but similar outcomes to easier MV repairs. Authors have concluded that, with robotic technology, patients’ outcomes have not worsened despite having more complex disease [13]. Similarly, Fujita et al. presented 9-year data on 335 patients undergoing either robotic or mini-thoracotomy approach. The average score for repair complexity was significantly higher in the robotic group; particularly, in the robotic group, a complex repair (Category 2) was performed in 20% of the patients, and a most complex repair (Category 3) was performed in 17% of the patients, whereas a complex repair was performed in 12% of the patients and a most complex repair was performed in 4% of the patients in the minithoracotomy group [14]. In our experience, we have confirmed that case complexity increased with experience. Globally, simple MV repair was performed in 53% of patients, complex in 37%, and most complex in 10%, with an overall MV repair rate of 100% and no conversion to MV replacement. Moreover, a significant increase in MV repair complexity was observed, with a higher percentage of Category 2 and 3 MV repairs in Group 2 (36% in Group 1 vs. 52% in Group 2, *p* = 0.001). We feel that these excellent results in complex MV disease treatment are related to the unique characteristics of the robotic system. The “classic” minithoracotomy MV surgery may narrow the repertoire of mitral repair techniques used: shafted instruments have limited freedom of movement at the tissue interface, and a static left atrial (LA) retractor lacks versatility in visualizing the various components of the mitral apparatus. In the authors’ opinion, the robotic platform overcomes the limitations; first, enhanced surgical dexterity is allowed, facilitating precise movements of instruments in the closed chest and avoiding the fulcrum effect characteristic of long shafted endoscopic instruments. Second, the high-resolution 3D camera combined with the flexibility of a dynamic LA retractor affords an unsurpassed view of the valve and its components.

## 5. Conclusions

Robotic-assisted MV repair is a safe procedure associated with excellent short and long-term results. Experience is a key element to overcome the limitations of this technology. Finally, the robotic platform could improve results in difficult MV repair.

## Figures and Tables

**Table 1:** Preoperative data.

	All (144 PTS)	Group 1 (39 PTS)	Group 2 (105 PTS)	*p* Value
Age, years (mean ± SD)	58 ± 10	56 ± 10	59 ± 10	ns
Male, *n* (%)	131 (91)	36 (92)	95 (90.5)	ns
Hypertension, *n* (%)	78 (54)	22 (56)	56 (53)	ns
Diabetes Mellitus, *n* (%)	3 (2.5)	1 (2.5)	3 (2.5)	ns
Extracardiac Arteriopathy, *n* (%)	3 (2)	2 (5)	1 (1)	ns
COPD, *n* (%)	6 (4)	1 (2.5)	5 (4.5)	ns
EuroSCORE II (mean ± SD)	0.8 ± 0.5	0.7 ± 0.6	0.8 ± 0.6	ns
STS Score (mean ± SD)	0.6 ± 0.3	0.6 ± 0.2	0.6 ± 0.4	ns
MV pathology				
Barlow disease, *n* (%)	13 (9)	3 (7)	10 (9)	ns
Posterior Leaflet pathology, *n* (%)	136 (94)	35 (90)	101 (96)	ns
Anterior Leaflet pathology, *n* (%)	10 (7)	1 (2.5)	9 (8.5)	0.03
LVEF > 55%, *n* (%)	141 (98)	37 (95)	104 (99)	ns

PTS, patients; SD, standard deviation; COPD, chronic obstructive pulmonary disease; STS, Society of Thoracic Surgeons; MV, mitral valve; LVEF, left ventricular ejection fraction.

**Table 2:** Operative Data.

	All (144 PTS)	Group 1 (39 PTS)	Group 2 (105 PTS)	*p* Value
Transareolar approach, *n* (%)	14 (10)	4 (10)	10 (9.5)	ns
Jugular-femoral venous cannulation, *n* (%)	72 (50)	39 (100)	33 (31)	<0.001
Femoral venous cannulation, *n* (%)	72 (50)	0 (0)	72 (69)	< 0.001
Femoral arterial cannulation, *n* (%)	143 (99.3)	39 (100)	104 (99)	ns
Axillary arterial cannulation, *n* (%)	1 (0.7)	0 (0)	1 (1)	ns
Endoclamp, *n* (%)	28 (19)	28 (72)	0 (0)	<0.001
External clamp, *n* (%)	116 (80)	11 (28)	105 (100)	<0.001
CPB time, min (mean ± SD)	128 ± 40	155 ± 44	121 ± 36	0.002
Cross-clamp time, min (mean ± sd)	78 ± 42	112 ± 25	68 ± 39	<0.001
Conversion to sternotomy, *n* (%)	1 (0.7)	0 (0)	1 (1)	ns
Triangular resection PL, *n* (%)	68 (60)	16 (41)	70 (67)	0.002
Quadrangular resection PL, *n* (%)	35 (24)	4 (10)	31 (29)	0.01
Commissuroplasty, *n* (%)	5 (3.5)	0 (0)	5 (5)	ns
Sliding plasty, *n* (%)	50 (35)	3 (7)	47 (45)	<0.001
Neochordae, *n* (%)	39 (27)	24 (65)	15 (14)	<0.001
Flexible band, *n* (%)	144 (100)	39 (100)	105 (100)	ns

PTS, patients; CPB, cardiopulmonary bypass; SD, standard deviation; PL, posterior leaflet.

**Table 3:** Postoperative data.

	All (144 PTS)	Group 1 (39 PTS)	Group 2 (105 PTS)	*p* Value
In-hospital Mortality, *n* (%)	1 (0.7)	0 (0)	1 (1)	ns
AMI, *n* (%)	1 (0.7)	1 (2.5)	0 (0)	ns
Stroke major, *n* (%)	1 (0.7)	1 (2.5)	0 (0)	ns
Revision for bleeding, *n* (%)	4 (2.5)	0 (0)	4 (4)	ns
Blood transfusion, *n* (%)	24 (16)	13 (33)	11 (10)	<0.001
ICU stay, hours median (IQR)	24 (48)	24 (20–30)	27 (24–49)	ns
Ventilation time, hours–median (IQR)	6 (1–15)	7 (5–19)	6 (1–15)	ns
In-Hospital stay, days–median (IQR)	8 (7–12)	9 (8–12)	8 (7–12)	ns
MR > 1+, *n* (%)	0 (0)	0 (0)	0 (0)	ns
LVEF > 55%, *n* (%)	139 (96)	36 (92)	103 (98)	ns

PTS, patients; AMI, acute myocardial infarction; ICU, Intensive Care Unit; IQR, interquartile range; MR, mitral regurgitation; LVEF, left ventricular ejection fraction.

## Data Availability

The data presented in this study are available on request from the corresponding author due to privacy.
